# Germline *HOXB13* mutation p.*G84E* do not confer an increased bladder or kidney cancer risk in polish population

**DOI:** 10.1186/s13053-021-00208-8

**Published:** 2022-01-04

**Authors:** Elżbieta Złowocka-Perłowska, Aleksandra Tołoczko-Grabarek, Jan Lubiński

**Affiliations:** Department of Genetics and Pathology, International Hereditary Cancer Center, Pomeranian Medical University, Szczecin, Poland

**Keywords:** P.*G84E* mutation, *HOXB13*

## Abstract

**Introduction:**

The role of *HOXB13* in bladder and renal tumorigenesis is unclear. Our goal was to determine the prevalence of *HOXB13* p.*G84E* mutation in bladder and kidney cancer patients from Poland.

**Materials and methods:**

1418 patients with bladder cancer and 813 cases with kidney cancer and 4497 controls were genotyped for *HOXB13* p.*G84E.*

**Results:**

p.*G84E* mutation of *HOXB13* gene was detected in three of 1418 (0.2%) bladder cancer cases and in six of 4497 controls (odds ratio [OR], 1.6; 95% CI 0.39–6.36; *p* = 0.8). Among 813 kidney cancer cases *HOXB13* mutations was reported in three patients (0,4%) (odds ratio [OR], (OR = 2,8; 95% CI 0.69–11.11; *p* = 0.3). In cases with mutations in the *HOXB13* gene, the family history of cancer was negative.

**Conclusion:**

*HOXB13* mutation was not associated with bladder or kidney cancer**.** Mutation p.*G84E* in *HOXB13* seem not to play a role in bladder and kidney cancer development in Polish patients.

## Introduction

In 2017, cancer caused 26% of deaths among men and 23% among women in Poland. In men one of the most common cancers of the urinary tract is bladder cancer - 7% and kidney cancer - 4%. In women, bladder cancer accounts for less than 1% and kidney cancer for 2.5% of all cancer cases [1].

The exact molecular mechanisms underlying the initiation and progression of bladder or kidney cancer remain largely unknown. High hereditary predisposition for bladder cancer is presents in 4% and for kidney cancer 5% [2, 3]. A number of mechanisms play a large role in the initiation of neoplastic transformation of bladder or kidney cancer, including: mutation of suppressor genes, activation of proto-oncogenes, abnormal and over-expression of oncogenes through amplification and deletion of some regions of chromosomes, and methylation. Thus the genetic basis leading to a better or worse prognosis for survival in cancer patients may be dependent on functional polymorphisms in genes such as genes responsible for tumor transformation, xenobiotic metabolism, oxidative stress, detoxification and DNA repair. Genes whose expression leads to the development of bladder and kidney cancer are being searched for. These are the so-called high penetration genes or so-called low penetration genes whose expression disturbs metabolism and may initiate the process of neoplastic transformation of various organs. To date, no genes with high penetration into bladder cancer or kidney cancer have been identified. The low penetration genes responsible for the metabolism of carcinogenic chemicals, repair and removal of DNA damage include: *XRCC1, XRCC3, NAT2, XPD, RAG1, hOGG1 GSTM, NAT2, XRCC3, GSTP1, RAD23B, ERCC2, ERCC5, ERCC1, XPC, GSTT1, ADPRT.* The genes encoding enzymes involved in the process of metabolic activation, detoxification and DNA repair are polymorphic and there are some structural variants of genes in the population associated with different activity of the enzyme they encode.

The *HOX* gene family belongs to the homeobox superfamily of transcription factors. HOX consensus binding sites were found in the p53 promoter [4]. A disorder of the cell cycle and mutations in the TP53 gene were identified in 50% of patients with bladder cancer [5, 6]. A mutation in the TP53 gene overexpresses the p53 protein and increases the risk of tumor progression [7]. It is noteworthy that the *HOXB13* gene is located on chromosome 17q, the loss of heterozygosity of which has been noted in cancer of the kidney, breast, ovary, colon and some haematological malignancies. In turn more copies of chromosomes have been observed in bladder cancer or neuroblastoma [8–15]. Altered expression of *HOX* genes may be important for oncogenesis and tumor suppression by influencing various pathways that promote tumorigenesis and metastasis. *HOX* and homeobox genes play an important role in the regulation of many processes including cell proliferation, differentiation, angiogenesis, receptor signaling, apoptosis and regulate transcription of target androgen receptor genes [16]. Abnormalities in the expression of the *HOX* gene have been reported in abnormal development and malignant neoplasms of breast, leukemia, prostate, cervix, ovary, bladder, kidney, neuroblastoma, lung and esophageal squamous cell carcinoma [17]. *HOX* genes showed pro-angiogenic properties through increased expression of CXCL1, FGF2, IL8 and VEGFA which suggests a role in tumor progression [18].

In Poland, mutations in *HOXB13* are the cause of 0.6% unselected cases of prostate cancer [19]. The bladder and prostate derive from the same embryological structure, the urogenital sinus [20]. To determine whether the *G84E* mutation in the *HOXB13* gene contributes to the development of bladder or kidney cancer in Poland, and to measure the impact of this variant on cancer risk and on survival, we genotyped 1418 unselected bladder patients and 813 unselected kidney cancer cases and 4497 healthy controls**.**

## Material

### Patients

This study includes 1418 unselected cases of urothelial bladder cancer (378 women and 1040 men) and 813 unselected patients with kidney cancer (357 women and 456 men) diagnosed at the Urology Hospital in Szczecin between 2000 and 2018. A total of 1518 incident cases of bladder cancer and 841 kidney cancer were identified during the study period. Of these, 1418 patients with bladder (93%) and 813 with kidney cancer (97%) accepted the invitation to participate in the study. All eligible patients provided written informed consent for a blood draw specifically for research purposes and for storage of the blood sample in an existing research biobank. Pathologic diagnosis of bladder and kidney cancer was confirmed by review of biopsies at a single central pathology laboratory in Szczecin, Poland. All cases were unselected for age, sex, smoking status and family history. The mean age of diagnosis for bladder cancer patients was 69 years (range 20–91) and was 63 years (range 19–85) of kidney cancer. Detailed information of smoking status was available for a subset of 1090 (77%) cases with bladder and 478 (59%) kidney patients (pack years). A family history was taken by the construction a pedigree questionnaire. A total of 46 patients with a family history of at least one bladder cancer in first or second degree relatives and 29 cases with a family history of at least one kidney cancer in first or second degree relatives were identified. The vital status and the date of death of all of the cases were requested from the Polish Ministry of the Interior and Administration in January 2021, which was obtained in February 2021. In total we collected data of death of 821 (58%) patients with bladder and 257 (31%) kidney cancer. The study was approved by the Ethics Committee of Pomeranian Medical University in Szczecin.

### Controls

The control group included 4497 cancer-free, population-based, adults from (the same genetically homogeneous population as the patients) of West Pomerania in Poland. In order to accurately estimate the frequency of the p.*G84E* mutation in *HOXB13* the two control groups were pooled. The first group consisted of 2604 cancer-free men with the range of 23–90 years old (mean age 61.2 years) unselected for family history. The second group consisted of 1893 cancer-free females aged 19–91 years (mean age 52.2 years) unselected for family history too. These controls are described in detail elsewhere, male controls [19] and female controls [21]. The allele frequencies for all variants in our control group were not dependent on age or sex, and the prevalence estimates of mutations in all genes were similar in younger and in older controls.

## Methods

DNA was extracted from peripheral blood for all cases and controls. The presence of the p.*G84E* was genotyped as described previously [19]. In brief, we assessed this variant by genotyping using a TaqMan assay (Life Technologies, Carlsbad, CA) in a LightCycler Real-Time PCR 480 System (Roche Life Science, Mannheim, Germany). All mutations were confirmed by Sanger sequencing using a BigDye Terminator v3.1 Cycle Sequencing Kit (Life Technologies), according to the manufacturer’s protocol. In all reaction sets, positive and negative controls (without DNA) were used.

### Statistical analysis

#### Odds ratios

The prevalence of *HOXB13* allele was compared in bladder cancer cases and in controls, singly and in combination. Odds ratios were generated from two-by-two tables and statistical significance was assessed using the Fisher exact test where appropriate. Odds ratios were generated by patient subgroups defined by the presence or absence of the p.*G84E* mutation also as estimates of relative risk and additionally were adjusted for age, sex and pack-years of smoking by multiple logistic regression.

#### Ethical statement

The study was performed in accordance with the principles of the Declaration of Helsinki. All patients and controls provided written informed consent.

## Results

### Bladder cancer

Of the 1418 bladder cancer patients enrolled in the study, three (0.2%) carried a *HOXB13* mutation p.*G84E* (OR = 1.6; 95% CI 0.39–6.35; *p* = 0.8) (Table 1). A *HOXB13* mutation was seen in the group of 1040 affected men (0.2%) and in 378 affected women (0.3%). We collected information about smoking from 1044 patients with bladder cancer including 121 (12%) nonsmokers and 923 (88%) smokers. We found no association between smoking and mutation rates. The p.*G84E* mutation was noticed in one smoker (0.1%), in a nonsmoker (0.8%) and data from the third person was missing. In the *HOXB13* gene none of the tested mutation were found in 46 family cases of bladder cancer in first- and/or second-degree relatives. Two patients with cancer cases and the *HOXB13* p.*G84E* mutation died up to a year after diagnosis and one was still alive by February 2021.

**Table 1:** Effect of *HOXB13* mutations on bladder cancer risk

Mutation subjects	Number of carriers/total (frequency %)	OR	95% CI	***p***-value
*HOXB1* p.*G84E*				
Controls	6/4497 (0.1)	1.0		
Cases	3/1418 (0.2)	1.6	0.39–6.35	0.8

### Kidney cancer

In total, 813 kidney cancer cases and 4497 controls were genotyped for *HOXB13* mutation p.*G84E.* The mutation p.*G84E* was found in three (0.4%) of the cancer cases and in six (0.1%) of the controls (OR = 2.8; 95% CI 0.69–11.11; *p* = 0.3) (Table 2**)**. The *HOXB13* mutation was observes only in the group of 350 women (0.9%). As expected, the exposure to tabacco smoke was not an important risk factor in relationship between smoking and mutation frequency. Information about smoking we collected from 478 kidney cancer patients including 188 (39%) nonsmokers and 290 (61%) smokers. One p.*G84E* mutation was observed in a smoker (0.34%), in one nosmoker (0.54%) and we had no data from a third person with mutation. We did not register any investigated mutation in 29 family cases with kidney cancer in first- and/or second-degree relatives. We noticed that two patients with kidney cancer were still alive to February 2021 and the third one died two years after being diagnosed with kidney cancer.

**Table 2:** Effect of *HOXB13* mutations on kidney cancer risk

Mutation subjects	Number of carriers/total (frequency %)	OR	95% CI	***p***-value
*HOXB1* p.*G84E*				
Controls	6/4497 (0.1)	1.0		
Cases	3/813 (0.4)	2.8	0.69–11.11	0.3

## Discussion

In this study, we found no impact of the p.*G84E* mutation in the *HOXB13* gene on bladder or kidney cancer. We examined an unselected cohort of 1418 bladder cancer, 813 kidney cancer cases and 4497 controls and the p.*G84E* mutation was rare in the general population (0.1%). In the present study we noticed that mutation in *HOXB13* gene were observed in three (0.2%) unselected bladder cancer patients and three (0.4%) unselected renal cancer patients. The statistical power of the study is 35,5% for bladder cancer and 93,2% for kidney cancer.

Based on a review of the evidence in the literature, there are several studies that investigate the effect of the p.*G84E* mutation in the *HOXB13* gene in unselected bladder and kidney cancer cases, but they are based on small study cohorts. The investigators Beebe-Dimmer et al. genotyped 208 men with bladder and 137 men with kidney cancer patients and suggested the association between p.*G84E* and bladder cancer. The risk of a p.*G84E* mutation in bladder cancer was almost significant (*p* = 0.06). Their studies excluded women [22]. In another study by Hoffmann et al. followed 335 patients with bladder and 303 cases with kidney cancer. They observed similar to Beebe-Dimmer et al. that in bladder cancer the mutation was almost statistical significant (*p* = 0.06). Hoffmann et al. found an increased risk of p.*G84E* mutation in kidney cancer (*p* = 0.03) [23]. Okuda et al demonstrated that *HOXB13,* like the tumor suppressor gene, plays a role in tumorigenesis of human renal cell carcinoma and progression by participating in the apoptotic pathway [24]. Marra et al. in a small group of 86 bladder cancer samples over expression and more aggressive phenotype of *HOXB13* was observed in the muscle invasive with non-muscle invasive tumors [25].

In the literature there are several studies on mutation in the *HOXB13* gene and association with different cancers. Some studies have confirmed that mutation in the *HOXB13* gene was associated with early-onset prostate cancer men, cases with significantly elevated PSA and family history of prostate cancer [18, 26–28]. Additionally, abnormal expression of *HOXB13* has been detected in the development of leukemia, non-Hodkin’s Lymphoma, oral squamous cell carcinoma, glioma, colon, stomach and genitourinary cancers [17, 29–36]. Ovarian, cervical and endometrial cancers overexpress *HOXB13* [29, 37]. Moreover a link with melanoma cancer has been suggested and it predicts breast cancer recurrence and response to tamoxifen treatment [23, 37–39].

There are several strengths of our study including the large number of patients and controls, and the sampling of incident cases, unselected for age or family history. Cases and controls are all residents of Szczecin. There is no reason to believe that age, gender or smoking behavior are important confounders of the observed association.

In conclusion, this study reveals that the p.*G84E* mutation in the *HOXB13* gene does not seem to play a role in development of bladder or kidney cancer. Our results indicate that testing p.*G84E* mutations is unlikely to be relevant for the identification of individuals at risk of bladder or kidney cancer, at least in the Polish population.

### Limitations

Our study was not without various limitations including a lack information about all patients.

## Data Availability

Our data contain potentially sensitive information therefore we have not included it with our manuscript. Those who would like to request access to data may contact Melissa Sidhu at the Research Ethics Board of Women’s College Hospital by calling (416) 351–3732 × 2723 or email ac.latipsohcw@uhdis.assilem.
